# Cancer drug response prediction with surrogate modeling-based graph neural architecture search

**DOI:** 10.1093/bioinformatics/btad478

**Published:** 2023-08-09

**Authors:** Babatounde Moctard Oloulade, Jianliang Gao, Jiamin Chen, Raeed Al-Sabri, Zhenpeng Wu

**Affiliations:** School of Computer Science and Engineering, Central South University, Changsha 410083, China; School of Computer Science and Engineering, Central South University, Changsha 410083, China; School of Computer Science and Engineering, Central South University, Changsha 410083, China; School of Computer Science and Engineering, Central South University, Changsha 410083, China; School of Computer Science and Engineering, Central South University, Changsha 410083, China

## Abstract

**Motivation:**

Understanding drug–response differences in cancer treatments is one of the most challenging aspects of personalized medicine. Recently, graph neural networks (GNNs) have become state-of-the-art methods in many graph representation learning scenarios in bioinformatics. However, building an optimal handcrafted GNN model for a particular drug sensitivity dataset requires manual design and fine-tuning of the hyperparameters for the GNN model, which is time-consuming and requires expert knowledge.

**Results:**

In this work, we propose AutoCDRP, a novel framework for automated cancer drug–response predictor using GNNs. Our approach leverages surrogate modeling to efficiently search for the most effective GNN architecture. AutoCDRP uses a surrogate model to predict the performance of GNN architectures sampled from a search space, allowing it to select the optimal architecture based on evaluation performance. Hence, AutoCDRP can efficiently identify the optimal GNN architecture by exploring the performance of all GNN architectures in the search space. Through comprehensive experiments on two benchmark datasets, we demonstrate that the GNN architecture generated by AutoCDRP surpasses state-of-the-art designs. Notably, the optimal GNN architecture identified by AutoCDRP consistently outperforms the best baseline architecture from the first epoch, providing further evidence of its effectiveness.

**Availability and implementation:**

https://github.com/BeObm/AutoCDRP.

## 1 Introduction

An important organ can be damaged or even destroyed by cancer, a term used in the medical field to describe a group of diseases caused by the uncontrolled growth of abnormal cells. Cancer can be cured through appropriate drug treatments. However, differences in sensitivity to the same treatment can be found in many patients with the same type of cancer due to the diverse genetic backgrounds, lifestyles, and environments of individuals (Castiblanco and Anaya 2015), which makes cancer drug–response prediction (CDRP) challenging. In fact, organisms acquire their characteristics through complex interactions between their genetic background and environmental factors that influence their ability to metabolize a specific drug. As the exact relationship between genetic backgrounds and the effects of the environment is still unclear, it remains challenging to accurately diagnose cancer drug response using genetic information (Friedman *et al.* 2015). Traditional methods for CDRP are mostly based on *in vivo* animal experiments and *in vitro* drug screening, but these methods are costly and laborious (Pandiyan and Wang 2022).

With the increased availability of molecular data from cancer patients, several large-scale drug–response projects have been developed, including the cancer cell line encyclopedia (CCLE) (Barretina *et al.* 2012) and genomics of drug sensitivity in cancer (GDSC) (Iorio *et al.* 2016). These projects provide gene expression profiles and drug–response profiles for hundreds of cancer cell lines (artificial patients). As a result, a variety of machine-learning-based computational methods have been proposed for CDRP. Earlier methods formulated CDRP as a matrix factorization problem based on similarity involving the generation of a matrix consisting of drugs and cancer cell lines. For example, Zhang *et al.* (2018) predict anti-cancer drug responses of cell lines using a hybrid interpolation weighted collaborative filtering approach based on cell line and drug similarity. Liu *et al.* (2018) predict anti-cancer drug responses of cell lines by formulating drug–response prediction as a recommender system problem and using a neighbor-based collaborative filtering method. The approach integrates cell-line similarity networks and drug similarity networks, removing global effects and shrinking similarity scores to predict drug responses. Guan *et al.* (2019) propose a method that predicts anti-cancer drug response in cell lines, using weighted graph regularized matrix factorization. The method specifies drug similarity and cell-line similarity matrices using a *p*-nearest neighbor graph and performs matrix factorization to generate latent matrices for drugs and cell lines. However, matrix factorization based on similarity methods for CDRP has limitations including data sparsity, and overfitting, due to the fact that drug–response functions of cell lines are non-linear and complicated (Chen and Zhang 2021). To tackle this issue, many network methods (Liu *et al.* 2019, Peng *et al.* 2023) have been extensively employed for predicting cancer drug response. Liu *et al.* (2019) introduce tCNNS, a method that uses convolutional neural networks to predict drug response. It extracts drug features from the simplified molecular-input line-entry system (SMILES) strings and cell-line features from genetic attribute vectors using convolution layers and combines them with a fully connected layer. Peng *et al.* (2023) predict drug response in cancer patients using molecular fingerprints as engineered drug features and graph convolutions to generate a heterogeneous network. However, the method’s assumption that similar cell lines have similar drug responses may not hold true due to genetic variations, and the use of engineered drug features may not fully capture biological complexity, making accurate prediction difficult. Drugs can be naturally represented as a graph as it has unique topological structures, such as edges (chemical bond) and nodes (atoms).

Over the past few years, graph neural networks (GNNs) have gained tremendous popularity in bioinformatics, especially in molecular structure representation learning where they have proven to produce excellent results (Nguyen *et al.* 2021a, b, Chu *et al.* 2023). For example, Nguyen *et al.* (2021a) proposes a deep learning-based method called GraOmicDRP to integrate the graph representation of drugs and multiple-omic data of cell lines for drug–response prediction. The proposed method achieves higher performance by combining informative data, such as transcriptomic data with other omic data. Chu *et al.* (2023) proposes a deep learning model, GraTransDRP, which utilizes a graph transformer to extract drug representation more efficiently and combines it with 1D convolutional neural networks to learn cell-line features. The model outperforms state-of-the-art methods, including GraOmicDRP. However, these methods are handcrafted models. They require manual design and fine-tuning of the hyperparameters for the GNN models, which is time-consuming, error-prone, and requires expert knowledge.

Recently, there has been a lot of attention given to graph neural architecture search (GraphNAS), which is used to automatically find the best neural architecture for graph representation learning task (Chen *et al.* 2022, Oloulade *et al.* 2022, Al-Sabri *et al.* 2023). The objective of GraphNAS, given a search space *S*, and a validation dataset *V*, is to select the best architecture $M^{*}\in S$ that optimizes an evaluation performance $F(M^{*})$. As evaluating the performance of architectures is a time-consuming process and the search space contains millions of architectures, existing frameworks typically stop the search process based on a predefined computation budget (Chen *et al.* 2022, Al-Sabri *et al.* 2023). There is a disadvantage to this strategy in that it limits the rate of exploration of the search space by the search algorithm. This reduces the chances of the GraphNAS framework acquiring the optimal architecture. For example, the search space used by Chen *et al.* (2022) contains over $10^{7}$ architectures but the search algorithm only explores 2000 architectures. Because the exploration of search algorithms is limited to 0.2%, the likelihood of obtaining the global optimal GNN architecture is greatly reduced.

In this work, we present AutoCDRP, a framework for automatically learning drug structure representations using surrogate modeling. In AutoCDRP, a small set of architectures is sampled using a controlled stratified random sampling algorithm. The controlled stratified random sampling method is utilized to obtain GNN architectures uniformly from the search space, which ensures that the sampled architectures are representative of the overall architecture space, reduces the bias and variance in the sampling process, and allows efficient exploration of a large search space. The sampled architectures are evaluated, and a set of (architecture-performance) training pairs is created, which is used to train the surrogate model and predict the performance of every architecture in the search space. Finally, AutoCDRP selects the architecture with the most optimal evaluation performance out of the top-*k* predicted architectures. To ensure the high performance of the surrogate model in AutoCDRP, we carefully design the architecture and training process of the surrogate model in AutoCDRP, using a two-layer GNN and a batch loss function to handle different graph topologies and mitigate the effect of outliers. We validate the performance of the surrogate model on independent test sets and its generalization capability through extensive experiments and comparison with state-of-the-art methods. By using this method, we resolve the challenge of manually designing GNN for CDRP. Additionally, unlike the previous GraphNAS methods that were limited in their exploration of the search space due to computation cost, AutoCDRP can quickly evaluate a large number of architectures using the surrogate model, which increases the probability of finding the optimal solution. Our contributions are summarized as follows:

1. We propose a significant contribution to the field by introducing the first framework, AutoCDRP, that automates the design of GNN architectures for CDRP, eliminating the need for manual GNN design for CDRP.
2. AutoCDRP uses a surrogate model to explore all architectures in the search space, thereby increasing the exploration rate and probability of finding the optimal solution. Furthermore, a new search space is designed in AutoCDRP for predicting cancer drug response, which includes more recent GNN architecture designs.
3. We also demonstrate the effectiveness of AutoCDRP through extensive experiments. The results of the experiments show that architectures designed by AutoCDRP outperform state-of-the-art architectures.

## 2. Materials and Methods

### 2.1 Datasets

To test our model, we performed experiments on two datasets, GDSC (Yang *et al.* 2013) and CCLE (Barretina *et al.* 2012) datasets. (i) GDSC is a large-scale drug sensitivity screening project, which evaluated anti-cancer drugs on hundreds of cancer cell lines and gathered omics and drug–response data. We use GDSC version 6.0, which provides the half maximal-inhibitory concentration (IC50) values corresponding to a response score of drug–cell pairs across 250 drugs and 1074 cell lines. By comparison, Menden *et al.* (2013) use GDSC version 2.0, which contains 131 drugs and 639 cell lines. Moreover, cancer cell lines are described by their genetic characteristics i.e. omics data, such as copy number variants and mutations state, and drugs are described by their names and the compound id (CID) used as reference numbers to extract more information from other databases. We collect the molecular structure of the drugs from PubChem (Kim *et al.* 2019). (ii) CCLE (Barretina *et al.* 2012) provides comprehensive genomic and pharmacological data from human cancer cell lines. The dataset reports experimental information, such as drug target, dose, log(IC50), and effective area drug–cell pairs across 24 drugs and 1036 cell lines. In this study, we use log(IC50) as a measure of sensitivity.

### 2.2 Preprocessing

Following Liu *et al.* (2019), we only selected drugs with known IC50 values from the datasets. The SMILES strings of all drugs were downloaded from PubChem and compiled into a molecular graph where the atoms represented the nodes, and edges were denoted using bonds. The attribute of each atom node was represented as a 78D feature vector. We removed cell lines that lacked any type of omics data and drugs that shared the same CID in PubChem. We also excluded drugs without a PubChem ID in the GDSC database.

After processing the GDSC dataset, we collected a dataset containing 172 114 drug–cell pairs across 223 drugs and 948 cell lines. Considering all the 223×948=211 404 drug and cell-line interaction pairs, ∼18.6% of pairs were missing. The target value of each drug–cell pair was also normalized in a range (0, 1). Similarly, after preprocessing the CCLE dataset, we collected a dataset containing 11 104 drug–cell pairs across 23 drugs and 436 cell lines. Considering all the 24×436=10 464 drug and cell-line interactions, no drug–cell pair was missing. At the input stage, drugs were represented in canonical SMILES format, and the cell lines were represented by a binary 735D vector.

##  

### 2.3 Problem formulation

The task of CDRP is treated as a regression problem, aiming to predict the log normalized half maximal-inhibitory concentration [ln(IC50)] for given drug–cell pairs. To this end, a drug–response matrix R is constructed, which is a c×d matrix, where *c* and *d* represent the number of cell lines and drugs, respectively. Each entry $R_{i,j}$ represents the response score ln(IC50) of drug *j* for cell line *i*. The general task of CDRP is to determine a mapping function M that maps a drug–cell pair to its corresponding response score in the drug–response matrix R. In this study, the mapping function M is implemented as a GNN. The GNN model is composed of multiple convolution layers, each comprising four key components: aggregation, combination, activation, and normalization. The aggregation function is responsible for gathering information from neighboring nodes, thereby producing a vector representation that captures the local subgraph of the node. The combination function then merges this aggregated information with the node’s own feature representation, resulting in a new representation that incorporates both the local subgraph structure and the node features. Subsequently, the resulting representation undergoes an activation function and a normalization step, which contribute to enhancing the stability of the training process. Finally, a pooling function is employed to obtain a graph-level representation. By configuring different options for these components, a variety of GNN architectures can be created. To find the best-performing GNN architecture, we define a search space S consisting of all combinations of different component values, and a performance evaluation function F that measures the root mean squared error (RMSE). We then learn a surrogate function $\tilde{F}$ using a small set *T* of architectures to predict the performance $F(M_{i}\in S)$ of architectures in the search space, and use this to identify the architecture $M^{*}\in S$ with the best performance. The objective function is formulated as: where L is the loss function for the surrogate model $\tilde{F}$. Ω is a set of all possible approximation to F(M).


(1) $$\begin{array}{c} M^{*}=\underset{M\in S}{\mathrm{argmax}}\;\tilde{F}(M)\\ s.t\;\;\;\;\;\tilde{F}=\underset{\tilde{F}(M)\in \Omega }{\mathrm{argmin}}\;\sum_{M\in T}L(\tilde{F}(M),F(M)) \end{array},$$

**Figure 1.**
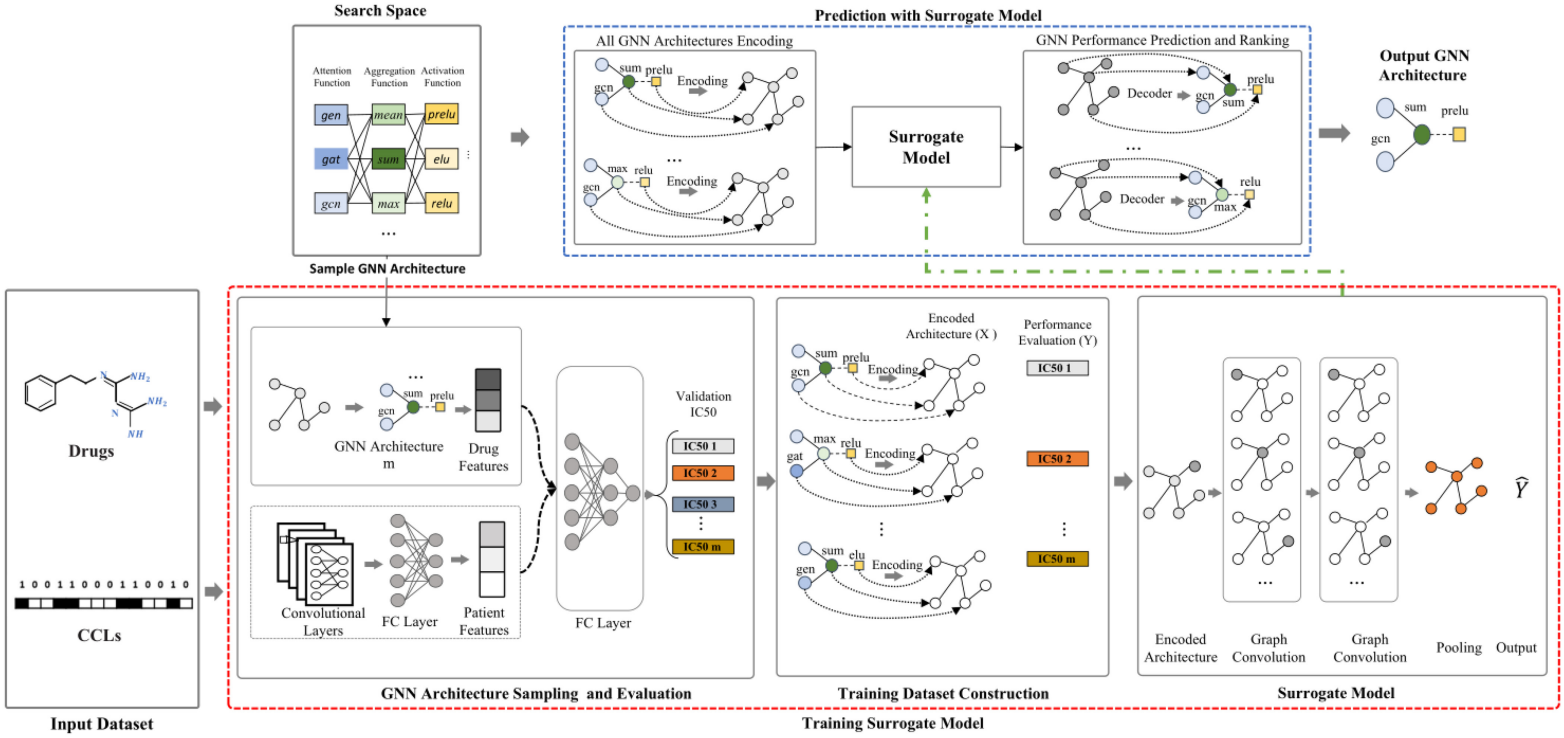
Surrogate-modeling-based graph neural architecture search framework. Sampled architectures are encoded as graphs with error labels. A surrogate model predicts architecture performance. Promising architectures are decoded, trained, tested, and compared. The best architecture is chosen as the target.

Based on the description of an architecture M given by an encoder model χ(⋅), we model the drug–cell pair response $R_{i,j}$. The first step is to sample and train |T| architectures from the search space. We obtain a set $T^{\prime }=\{(\chi (M_{1}),Y_{1}),(\chi (M_{2}),Y_{2}),\ldots ,(\chi (M_{\left|T\right|}),Y_{\left|T\right|})\}$, where $Y_{i}$ represent the performance evaluation of the architecture $M_{i}$. Following that, $T^{\prime }$ is used to train a surrogate model that predicts the performance evaluation of architectures in the search space. Finally, the top-*k* predicted architectures are then selected, trained, and validated, and the best one is chosen as the target architecture. The search efficiency can be increased by predicting the architecture’s performance because the surrogate model can provide a curated list of promising architectures after thoroughly evaluating all of them. As a result, the probability of finding the optimum solution increases significantly. Figure 1 shows the predictor-based framework, proposed to solve Equation (1).

### 2.4 Search space design

In this article, we explore a GNN search space that incorporates both architecture functions, such as aggregation and activation, and hyperparameters, such as dropout rate and learning rate. The designed search space in AutoCDRP is presented in Table 1. It consists of 12 design components, resulting in over $7\times 10^{9}$ possible architectures for a two-layers GNN. The designed search space can easily be extended to incorporate new dimensions that emerge in state-of-the-art models. To address the limitation of component choices, we employed two strategies. Firstly, we conducted a thorough literature review to identify a wide range of architectural components for GNNs in CDRP. This enabled us to select relevant features and enhance our framework, AutoCDRP, for accurate prediction. Secondly, we designed AutoCDRP to be adaptable, allowing for customization by researchers to experiment with different components and configurations beyond the initial choices presented in Table 1. This flexibility enables tailoring the framework to specific task requirements and datasets.

**Table 1.** Search space design.

Components	Choices
Aggregation type	Add, mean, max
Convolution type	GCNConv, GENConv, GINConv, SGConv, Linear
Dropout	False, 0.2, 0.4, 0.6
Activation function	Softplus, Linear, relu, PReLU, Sigmoid
Normalizer	BatchNorm, GraphNorm, none
Hidden dimension	16,64,128,256
Multi-head	1,2,4,6,8
Learning rate	1e-2, 1e-3, 1e-4, 5e-4
Weight regulation	1e-3, 1e-4, 5e-4
Optimizer	Adam, SGD
Loss criterion	CrossEntropyLoss, NllLoss
Pooling type	global_add_pool, global_max_pool

### 2.5 Surrogate model training

This section outlines the construction, training, and evaluation of the surrogate model in AutoCDRP.

#### 2.5.1 Surrogate model definition

The surrogate model is designed to provide an accurate evaluation of unobserved architectures. For this purpose, we define the surrogate model $\tilde{F}$ as a two-layer GNN based on GINConv (Xu *et al.* 2019) to fit the probability distribution between architectures and their performances. The surrogate model predicts performance based on input architecture graphs. The feature vector of an input graph node *v* at the *l*-th iteration, representing the *l*-th layer of the surrogate model can be defined as follow: where l∈[1,l] is the number of layers, $W_{l}$ is a trainable weight matrix in the *l*-th layer, $B_{l}$ is a bias, $B_{l}h_{v}^{l-1}$ is a self-loop activation for node *v*, and N(v) the neighborhood nodes of *v*. The representations of the nodes in the last convolution layer are averaged to obtain a graph-level representation followed by two fully connected layers and an output layer. To ease the effect of the outliers in loss calculation and prevent exploding gradients, a squared term is used when the absolute element-wise error falls below β=1 and the mean absolute term otherwise. Formally, for a batch size of *B*, the reduced loss $L_{\nabla }(X,Y)$ can be described as: where F(X) and $\tilde{F}(X)$ denote evaluation accuracy performance and predicted accuracy performance, respectively; with


(2) $$h_{v}^{\left(l\right)}=\mathrm{PreLU}(W_{l}⊙\sum (\{h_{u}^{l-1},\forall u\in N(v)\}),\gamma ),$$

(3) $$\gamma =B_{l}h_{v}^{l-1},h_{v}^{0}=x_{v},$$

(4) $$L_{\nabla }(F(X),\;\tilde{F}(X))=\mathrm{mean}({\left\{L_{\nabla }{}_{1},\ldots ,L_{\nabla }{}_{B}\right\}}^{T}),$$

(5) $$L_{\nabla }{}_{i}=\{\begin{array}{cc} 0.5\times {\left(F(X_{i})-\tilde{F}(X_{i})\right)}^{2}/\beta , & if\;|F(X_{i})-\tilde{F}(X_{i})|<\beta \\ |F(X_{i})-\tilde{F}(X_{i})|-0.5\times \beta , & \mathrm{otherwise} \end{array}.$$

The full hyperparameter settings for the surrogate model can be found in the experiments section.

#### 2.5.2 GNN architecture sampling and evaluation

In our study, we utilized the controlled stratified random sampling method to obtain GNN architectures uniformly from a large search space. This method involved fixing each candidate and randomly sampling a minimum number of GNN architectures containing the fixed candidate from the search space. By doing so, we ensured that the sampled architectures were representative of the overall architecture space, and reduced the bias and variance in the sampling process. This allows us to efficiently explore a large search space by sampling a smaller subset of architectures, which is particularly important in graph neural architecture search, where the search space is often very large. Given that there are *n* architectures that can be sampled and *q* options in the search space that can be selected, the sampling method consists of two steps. The first step is to determine a lower limit *m* of the number of GNN architectures *s* to be sampled for each candidate. Accordingly, assuming *n* is 300, *q* is 50, and the lower limit for the search space is m=6. The second step involves fixing each candidate and randomly sampling *m* GNN architectures containing the fixed candidate from the search space. Afterward, each architecture $M_{i}\in T$ is trained and its validation performance $F(M_{i})=Y_{i}$ is used as a ground truth architecture performance. By doing so, we ensured that the sampled architectures were representative of the overall architecture space, and reduced the bias and variance in the sampling process.

#### 2.5.3 Training dataset construction

After sampling and evaluating architectures, we encode them into directed acyclic graphs (DAGs), with the graph labels representing the architecture’s performance, then use the encoded graphs to train the surrogate model. The nodes in the encoded graph are represented by the specific options of a component, and the edges are defined by the direct interactions between components according to the data processing flow. Two scenarios are considered when building an edge between two nodes. The nodes representing two architecture components that are logically related based on data processing flow in the architecture will have a directed edge. The direction of the edge is determined by the data processing flow. It is also possible to put a direct edge between nodes A and B, representing components A and B, such that component A is a sub-module of component B. A component A can be considered a sub-module when it represents a parameter for another component. For instance, a hidden dimension is a sub-module of a convolution function. The direction of the edge indicates the dependency relationship between components A and B.

Based on the directed graph, the adjacency matrix can be determined and the node embeddings are initialized to one-hot vectors representing the component’s option for the architecture. For representing the node features, we use an embedded sparse vector with a size equal to the number of options in the search space.

### 2.6 Prediction with surrogate model

AutoCDRP utilizes the surrogate model to predict and rank the performance of architectures in a large search space efficiently. It encodes all architectures using the mentioned encoding method, obtaining a DAG representation. This encoded graph serves as input for the surrogate model, predicting architecture performance. The top-*k* predicted architectures are selected, evaluated, and the best one is chosen.

## 3. Results

### 3.1 Experiment setup

#### 3.1.1 Baseline methods

We compare the GNN architectures output by AutoCDRP with three groups of baselines. The first group comprises CNN-based models; we only use tCNNS (Liu *et al.* 2019), which is a state-of-the-art CNN method for CDRP. The second group comprises general GNN models, such as GCN (Kipf and Welling 2016), ARMA (Bianchi *et al.* 2022), and ChebNet (Defferrard *et al.* 2016). The third group includes state-of-the-art handcrafted GNN methods for CDRP, such as GraphDRP (Nguyen *et al.* 2021b), GraOmicDRP (Nguyen *et al.* 2021a), and GraTransDRP (Chu *et al.* 2023). In addition, we also compare AutoCDRP to random search. The models are compared using the Pearson correlation coefficient (PCC) and RMSE.

#### 3.1.2 Implementation settings

To train the surrogate model, we sample 800 architectures and select *k* = 100 best-predicted graph neural architectures for validation. During the process, we used 200 epochs, except for evaluating the best architecture, which used 300 epochs and assessed $10^{6}$ architectures. A graph isomorphism network (GIN) model with two layers is used as a surrogate model. We adapt our neural predictor for directed graphs by adjusting the GIN framework and modifying the GIN model, capturing dependencies while adhering to GIN principles and accounting for graph acyclicity. A random 80%/20% split is chosen for the dataset split. The surrogate predicts the RMSE of architectures as scalar vectors. When the element-wise error is below β=1, a squared term is used for loss calculation, and otherwise a mean absolute term. Adam optimizer updates weight, with a learning rate of 0.00001 and *L*2 regularization of 0.0005. The surrogate model trains 500 epochs in experiments. The experiments are implemented using Pytorch-Geometric library (https://github.com/rusty1s/pytorch_geometric) (Fey and Lenssen 2019)

### 3.2 Surrogate model performance

**Figure 2.**
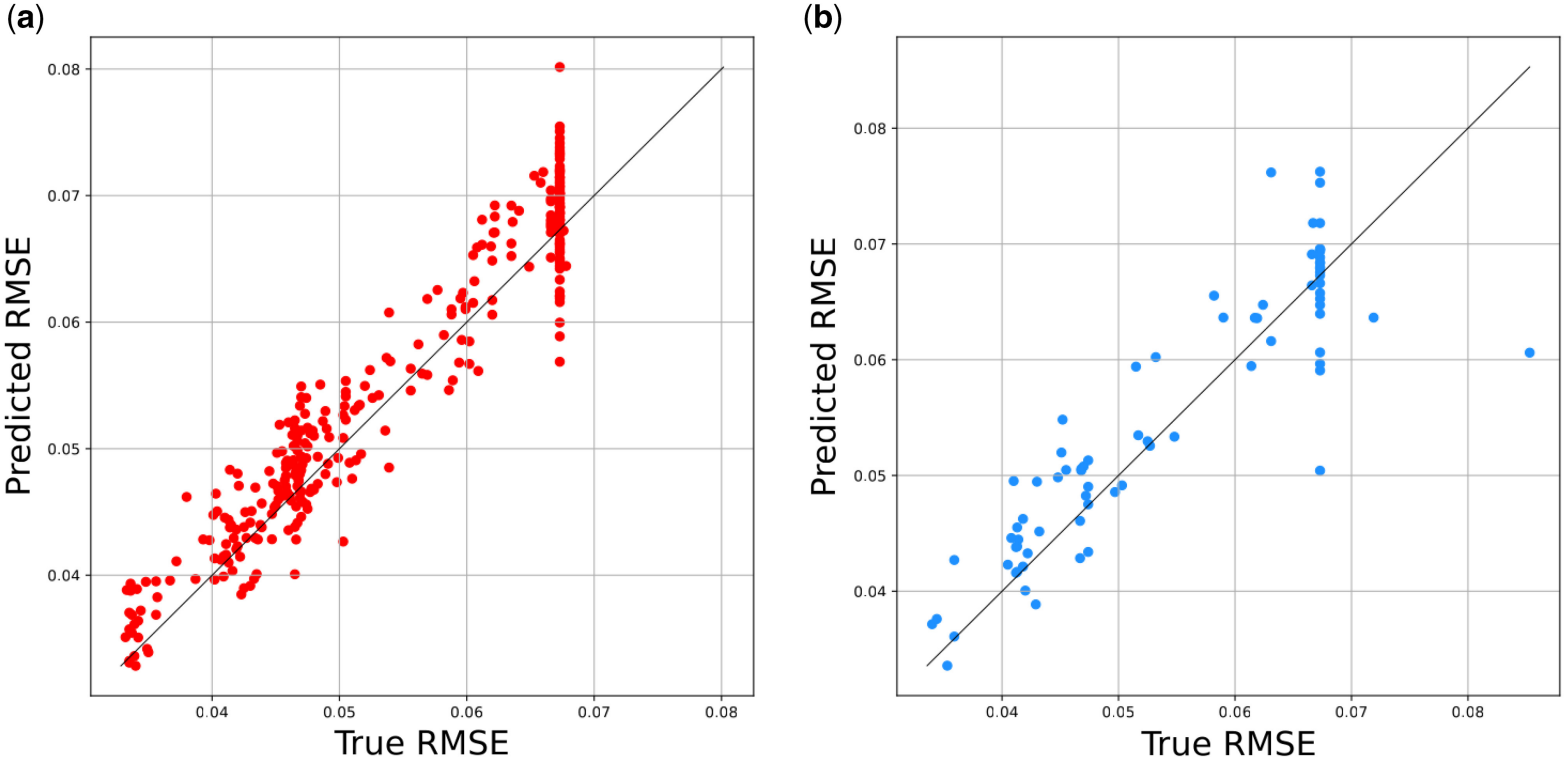
Surrogate model performance on the GDSC dataset. (a) Training samples and (b) test samples.

The surrogate model is evaluated using three metrics: RMSE, Pearson correlation, and Kendall tau correlation. A blind test was conducted on unseen cell lines and the results are presented in Fig. 2. For RMSE, Pearson correlation, and Kendall tau correlations, respectively, the surrogate model achieved performances of 0.0057, 0.9811, and 0.7895 on the training dataset. For RMSE, Pearson correlation, and Kendall tau correlation on the test dataset, the surrogate model achieved performance values of 0.0096, 0.7411, and 0.6352, respectively. Based on the evaluation of the training set, it can be concluded that the surrogate model accurately learns the distribution of training data, and the test set results indicate the surrogate model has the ability to predict the performance evaluation of architectures within the search space efficiently. This is due to the fact that AutoCDRP encodes GNN architecture using graphs, which can effectively capture the relationships between the components of a GNN architecture.

### 3.3 Comparison of overall performance

We examine and compare the GNN architectures produced by AutoCDRP with three groups of baseline models using PCC and RMSE metrics. The evaluation is performed on the same benchmark dataset and settings for all approaches. Two types of tests, the mixed test, and blind tests, were introduced. Table 2 presents the experiment results, with the best results indicated in bold.

**Table 2.** Performance comparison on the GDSC dataset in the mixed test experiment.

Method	Mix test				Blind tests with unseen drugs				Blind tests with unseen cell-lines			
	GDSC		CCLE		GDSC		CCLE		GDSC		CCLE	
	PCC	RMSE	PCC	RMSE	PCC	RMSE	PCC	RMSE	PCC	RMSE	PCC	RMSE
tCNNS	0.9160	0.0284	0.7720	0.0619	0.0617	0.0680	0.0226	0.0633	0.3490	0.0576	0.2027	0.0885
GCN	0.9118	0.0273	0.7858	0.0508	0.4467	0.0568	0.3769	0.0588	0.8230	0.0378	0.7279	0.0563
ARMA	0.9129	0.0272	0.7826	0.0511	0.3895	0.0577	0.0327	0.0636	0.8325	0.0370	0.7287	0.0562
ChebNet	0.9135	0.0271	0.7822	0.0511	0.3100	0.0630	0.3162	0.0600	0.8360	0.0364	0.7285	0.0563
GraphDRP	0.9300	0.0244	0.7834	0.0510	0.4292	0.0607	0.3687	0.0591	0.8302	0.0371	0.7292	0.0562
GraOmicDRP	0.9284	0.0252	0.7556	0.0542	0.4126	0.0632	0.0541	0.0660	0.8376	0.0364	0.7252	0.0575
GraTransDRP	0.9300	0.0244	0.7736	0.0520	0.5309	0.0482	0.0616	0.140	0.8405	0.0373	0.7257	0.0566

AutoCDRP_R	0.9318	0.0250	0.7813	0.0521	0.5189	0.0522	0.2942	0.0608	0.8588	0.0346	0.7277	0.0563
AutoCDRP	**0.9476**	**0.0224**	**0.7886**	**0.0500**	**0.5271**	**0.0422**	**0.3862**	**0.0581**	**0.8632**	**0.0333**	**0.7332**	**0.0562**

#### 3.3.1 Mixed test

In this experiment, we evaluate the performance of AutoCDRP using all available drugs and cell lines in the training step. This means, in the training step, all drugs and cell lines have been seen at least once. As GDSC provides the response only for 172 114 drug–cell pairs, we only consider 172 114 pairs. The data are shuffled and split into 80% as a training set, 10% as a validation set, and 10% as a test set. As shown in Table 2, GNN-based models outperform convolutional network-based model in both PCC and RMSE. More importantly, a model designed by our GraphNAS-based approach outperforms all handcrafted models for both PCC and RMSE. Moreover, considering only the handcrafted models, there is no absolute winner for both PCC and RMSE. Thus, the model designed by AutoCDRP is also strong.

#### 3.3.2 Blind tests with unseen drugs and cell lines

In previous experiments, drug–cell pairs were randomly picked to be in the training, validation, or testing datasets, which means that a specific drug or cell line could be part of the training and testing datasets. In practice, we would need to predict the response of drug–cell pair containing a new drug or new cell line. To ensure the efficacity of AutoCDRP in predicting unseen drugs or unseen cell lines, we design blind tests for drugs and cell lines. In the blind test for unseen drug experiment design, drugs are divided into the training, validation, and test datasets rather than the interaction pairs. In the blind test for unseen cell experiment design, cells are divided into the training, validation, and test datasets rather than the interaction pairs. The result for the test on unseen drugs and cell lines are also presented in Table 2. For blind drug and cell-line tests, prediction performance was not as good as for mixed test experiments of all models. This means that it is more difficult to predict cancer drug responses for new drugs or cell lines. However, it is obvious that AutoCDRP outperforms baseline methods in terms of both PCC and RMSE for bling tests for both unseen drugs and unseen cells.

#### 3.3.3 Random search

Besides surrogate-modeling-based search, a random search can also be used to solve the problem of graph neural architecture search for cancer drug prediction. While this baseline may seem simple, it is often difficult to outperform it in many cases. To show that AutoCDRP is efficient and effective, we do a supplementary experiment. In the first, we use random search instead of our search algorithm and note this method as AutoCDRP_R. As shown in Table 2, AutoCDRP_R outperforms all baselines in all experiments in terms of both PCC and RSME. The result also shows that AutoCDRP always outperforms AutoCDRP_R. Thus, even if a random search can provide competitive architectures, it cannot outperform AutoCDRP.

### 3.4 Statistical tests

**Figure 3.**
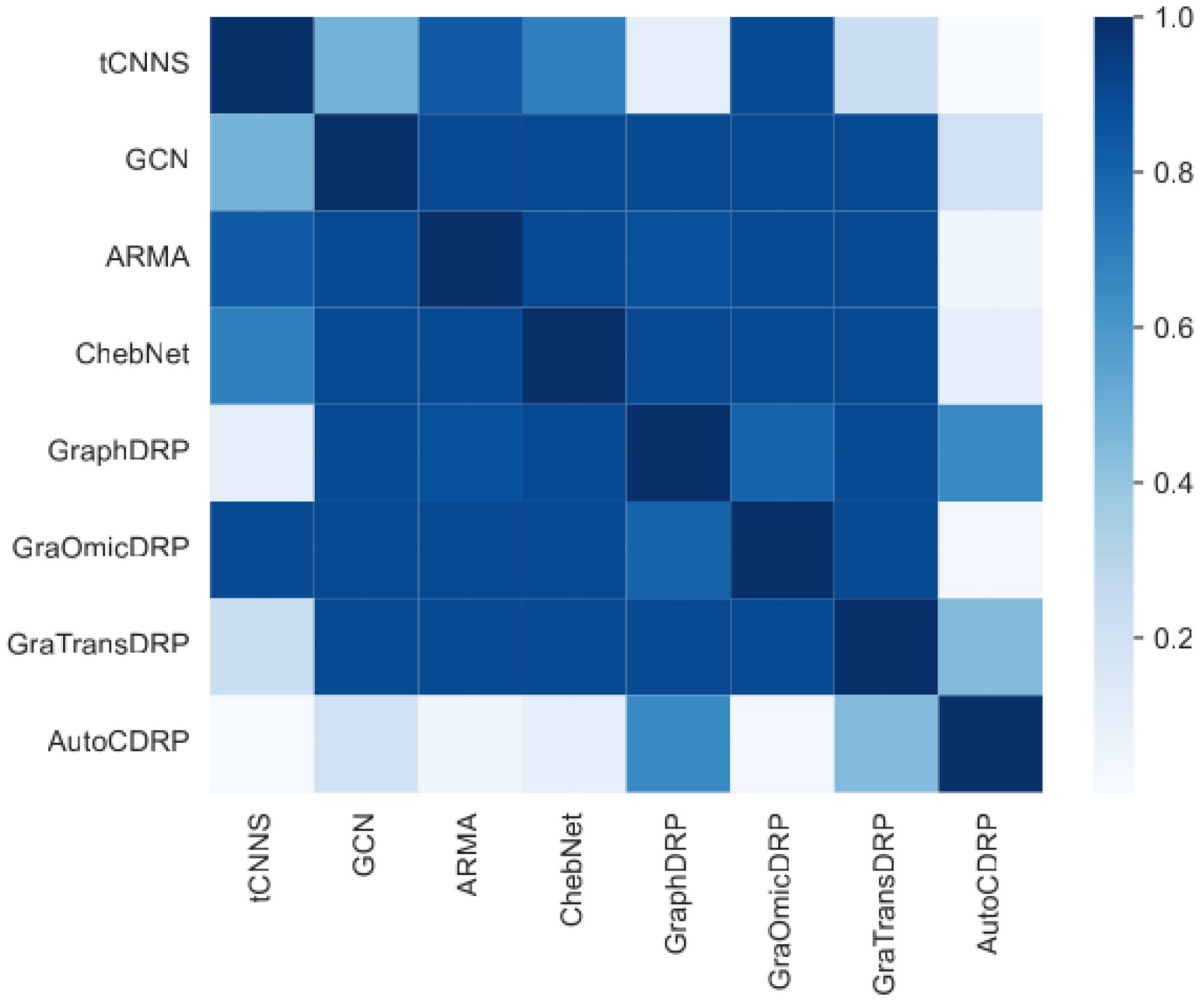
Nemenyi test scores. A low score means a significant difference between the performance of methods.

We apply non-parametric tests that are known to be suitable for comparing predictive models based on multiple data samples (Demsar 2006). Compared to the parametric tests, the non-parametric tests require fewer assumptions [1]. In this analysis, we use both GDSC and CCLE datasets and consider all experiments including mix test, blind test with unseen drugs, and blind test with unseen cell-lines experiments for each dataset. Thus, we make the analysis with height methods and six datasets. Firstly, Friedman test (Demsar 2006) is used to verify the hypothesis of equal performance among compared methods and the Bonferoni−Holm correction to compute family-wise *P*-values. The Friedman test revealed that the hypothesis of equal performance among compared methods should be rejected. Moreover, we computed family-wise *P*-values using Bonferoni−Holm correction. The family-wise *P*-values between AutoCDRP and baseline methods were $7.5224\times 10^{-06}$, 0.0014, 0.0021, 0.0046, 0.0133, 0.0391, and 0.0874 for tCNNS, GraOmicDRP, ARMA, ChebNet, GCN, GraTransDRP, and GraphDRP, respectively. It is revealed from the family-wise *P*-values that AutoCDRP shows significantly better performance over the baseline method with α=0.1. In addition, we make the Nemenyi test (Demsar 2006) to see how other baseline methods perform against each other. The Nemenyi test (Fig. 3) shows that every baseline method has significantly better performance over at most one other method while the proposed method significantly outperforms all baseline methods. More details about the statistical test can be found in the Supplementary Material.

### 3.5 Ablation study of the sampling method

We analyse the performance of the surrogate model employing various sampling methods, including controlled stratified random sampling, random, and uniform samplings. We train the surrogate model and evaluate it on both training and test datasets. As depicted in Table 3, the surrogate model can perform well on the training dataset for all experiments but performs best on the test dataset with the controlled stratified random sampling. This is because the controlled stratified random sampling reduces the bias and variance of the surrogate model by ensuring that the training dataset is representative of the population being studied. Additionally, it provides a diverse set of architectures to train on, which improves the generalization ability of the surrogate model. This shows that unlike random sampling and uniform sampling methods limit the predictability ability of the surrogate model.

**Table 3.** Performance results of the neural predictor in the ablation study of sampling methods on the train and test sets.

Metric	Controlled stratified		Uniform		Random	
	Train	Test	Train	Test	Train	Test
Pearson corr.	0.9811	0.7411	0.9782	0.7384	0.9792	0.7212
RMSE	0.0057	0.0096	0.0066	0.0124	0.0063	0.0135
Kendall tau	0.7895	0.6352	0.7882	0.6242	0.7798	0.5618.

### 3.6 Ablation analysis of the surrogate model

**Figure 4.**
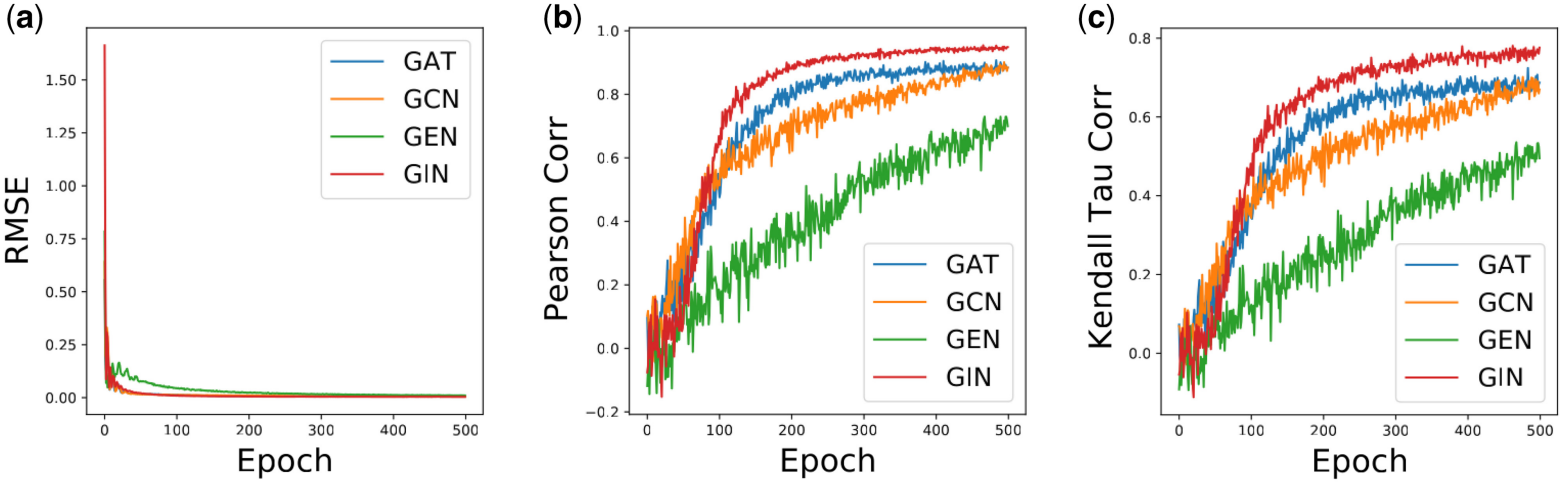
Surrogate models performance comparison. (a) RMSE, (b) Pearson correlation, and (c) Kendall tau correlation.

To prove the superiority of the surrogate model, we compare its ability to learn architecture performance distribution with popular models, such as GCN, GAT, and GEN. As shown in Fig. 4, the proposed surrogate model achieved better performance compared with other regressor models, showing its superiority. Compared with various regressor models, AutoCDRP can better fit the distribution relationship between GNN architecture and its real performance by using two-layer GIN to construct the surrogate model.

### 3.7 Convergence and average runtime analysis

**Figure 5.**
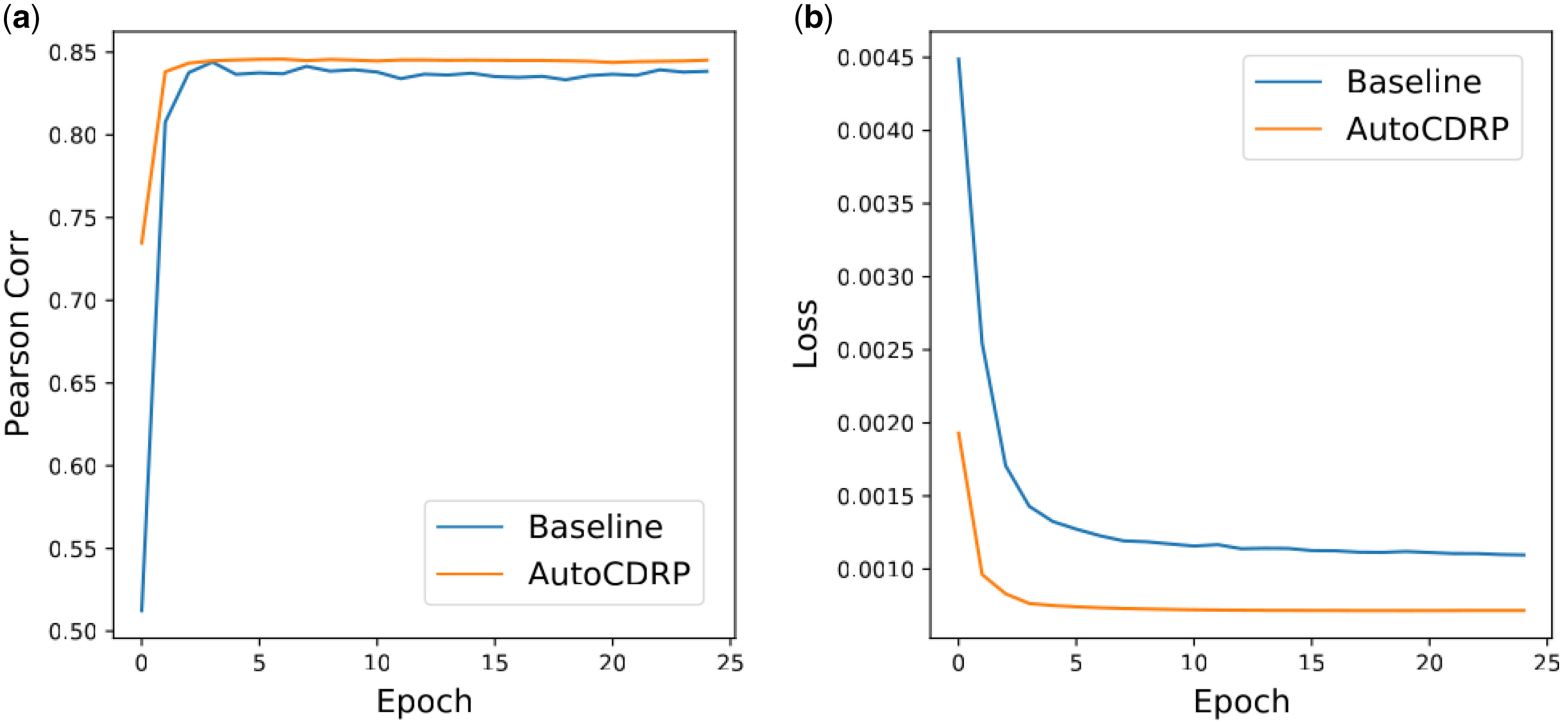
Pearson correlation and loss values for AutoCDRP and best baseline across training epochs 0 to 25. (a) Pearson correlation and (b) training loss value.

Regarding the convergence speed and time cost of the optimal architecture generated by AutoCDRP, we report the average Pearson correlation and loss values of the first 300 epochs in the training step. As shown in Fig. 5a, the *x*-axis represents the epoch number, and the *y*-axis represents the PCC. The optimal GNN generated by AutoCDRP performance is consistently better than the best baseline from the first epoch. The baseline needs about 23 epochs of training to output stable performance, while only about five epochs are needed for the optimal model generated by AutoCDRP. In the training phase, our GNN takes 128.70 s to complete a training epoch, which is 88.40 s shorter than the baseline. Therefore, the optimal performance is reached by AutoCDRP in a shorter time than the baseline because of the better convergence speed. The declining trend of loss values shown in Fig. 5b supports the above finding. The figure shows that the loss curve of the optimal GNN generated by AutoCDRP drops with a large gradient at the first epochs. In summary, the architecture generated by AutoCDRP can achieve better performance in a shorter time because of the good convergence speed.

## 4. Discussion

AutoCDRP shows promising results but also has some limitations that need to be considered. Firstly, the approach relies on a small set of GNN architectures, which may limit the diversity of architectures considered and lead to suboptimal performance. To address this limitation, future work could explore alternative methods for constructing the surrogate model, such as using a larger set of architectures or more sophisticated algorithms for model selection. Secondly, the surrogate model is trained on a limited set of hyperparameters, which may not generalize well to other hyperparameter settings and impact the performance of the approach. Lastly, the framework is focused on predicting drug response in cancer cells based on molecular features, which may not be directly applicable to other domains without modification. Despite these limitations, the AutoCDRP framework represents an important step toward a more efficient and effective graph neural architecture search for CDRP.

##  

In this work, we propose an end-to-end framework for CDRP, called AutoCDRP. AutoCDRP is an automated tool for learning drug molecular representations using surrogate modeling. Rather than training the architectures, it predicts their performance. With AutoCDRP, computing time is saved compared with manually designing architecture. Furthermore, AutoCDRP can explore a wide search space, which increases its efficiency and scalability. According to experiments conducted using two benchmark datasets, AutoCDRP architectures outperform state-of-the-art architectures for both PCCs and RMSEs in mixed tests, blind tests for unseen drugs, and blind tests for unseen cell lines.

Future research efforts could address the mentioned limitations and extend the proposed AutoCDRP framework to predict drug combination responses. Prior studies have demonstrated that predicting drug combination responses can significantly enhance cancer treatment outcomes (Chen *et al.* 2016, Li *et al.* 2023). Drawing inspiration from existing literature [28–31], it is worth exploring the incorporation of combination response data and examining ways to adapt the current framework to effectively handle the challenges associated with predicting drug combination responses. Therefore, we believe that incorporating combination response prediction into our framework will improve its practical utility and impact.

## Supplementary Material

btad478_Supplementary_Data

## Data Availability

The data underlying this article will be shared on reasonable request to the corresponding author.
