# Supplementary material for: Cancer drug response prediction with surrogate modeling-based graph neural architecture search
Source: Bioinformatics. 2023 Aug 9;39(8):btad478. doi: 10.1093/bioinformatics/btad478 (PMC10432359; doi:10.1093/bioinformatics/btad478)
Supplement: btad478_Supplementary_Data [file btad478_supplementary_data.pdf]

Cancer Drug Response Prediction With Surrogate Modeling-Based Graph Neural Architecture Search

Supplementary data

August 6, 2023

1 Controlled Stratified Random Sampling Algorithm

The proposed Controlled Stratified Random Sampling in AUTO-CDRP is presented in Algorithm 1

Algorithm 1 SamplingAlgorithm(S, n)

Input: S: search space
n: sampling size
Output: *samples* : sampled architectures

$TempDict \leftarrow S$
$ModelList \leftarrow \emptyset$
$q \leftarrow$ *total option in S*
if $n \ mod \ q == 0$ **then**
    $s \leftarrow n \backslash q$
else
    $s \leftarrow (n \backslash q) + 1$
end if
while $ModelList.size() < s$ **do**
    $ModelDict \leftarrow \emptyset$
    for func,opt in S **do**
        if $TempDict[func].size() == 0$ **then**
            $TempDict[func] \leftarrow opt$
        end if
        Select $ModelDict[func] \subset TempDict[func]$
        $TempDict[func].drop(ModelDict[func])$
    end for
    if $ModelDict \notin ModelList$ **then**
        $ModelList.append($ModelDict$)$
    end if
end while
$samples \leftarrow \emptyset$
for $function, option \in$S$.items()$ **do**
    for $component \in option.keys()$ **do**
        for $submodel \in ModelList$ **do**
            $model_{temp} \leftarrow submodel$
            $model_{temp}[function] \leftarrow option[component]$
            $sample.append(model_{temp})$
        end for
    end for
end for
return $samples[:n]$

2 Architecture Encoding Algorithm

Algorithm 2 present the proposed method for encoding a GNN architecture sampled from the search space into a direct acyclic graph.

Algorithm 2 Architecture Encoding(s, y)

Input:
s : *GNN architecture*
y : *validation performance of* s
q : *total number of options in the search space*
Output: X : *encoded architecture*

```
# Build initial node features
node_features ← ∅
for component, option ∈ s.items() do
    option_encoding ← one hot vector g ∈ ℝ^q
    node_features.append(option_encoding)
end for
#Construct edges
source ← ∅
target ← ∅
edge ← ∅
for comp, option ∈ s.items() do
    for u ∈ adjacentcomponentsofcomp do
        source.append(option.index)
        target.append(s[u].index)
    end for
    edge.append(source)
    edge.append(target)
end for
X ← GraphObject(node_features, edge, y, s)
Return X
```

3 Atom feature descriptions

The attribute of each atom node is represented as a 78-dimensional feature vector presented in Table 1.

Table 1: Atom feature descriptions

Features	Size	Description
Atom type	44	C, N, O, S, F, etc. (one-hot)
Degree	11	0–10 (one-hot)
Implicit valence	7	0–6 (one-hot)
Formal charge	1	Formal charge number (integer)
Radical electrons	1	Number of radical electrons (integer)
Hybridization	5	SP, SP2, SP3, SP3D, SP3D2 (one-hot or null)
Aromatic	1	Whether the atom is in an aromatic system (binary)
Hydrogens	5	0–4 (one-hot)
Ring	1	Whether the atom is in ring (binary)
Chirality	2	R, S (one-hot or null)

4 More Details About Proposed Search Space

Aggregate and Convolution and Functions

A message-passing scheme allows a node to acquire graph structure characteristics from its neighbor nodes through an aggregate function. The options for aggregation functions in the proposed search space include Add, $Mean$, and Max. As different neighbor node features have varying contributions to the representation of the central node in the graph, a convolution function is used to learn the different implications of each neighbor node in order to obtain a more accurate representation of the central node. There are many options for convolution functions in the proposed search space, such as $GCNConv$, $GENConv$, $GINConv$, $SGConv$, and $Linear$.

Multi-head and Hidden Dimension

As computing multiple independent convolution functions for the convolution operator is practical to stabilize the learning process, we add a multi-head component to our search space. Its options include 1, 2, 4, 6, and 8. In addition, since reducing and transforming the dimension of the original feature enhances the node's hidden representation, we also include a hidden dimension component in the search space. The hidden dimension value options are 16, 64, 128, and 256.

Normalization Function and Activation Function

In addition, we add a normalization function over the node features to enhance the expression ability of the model and an activation function to give the model a nonlinear fitting capability, which plays an important role in smoothing the hidden representation. Normalization functions available in the search space include $GraphNorm$, $BatchNorm$ and $None$, while activation functions include $sigmoid$, $relu$, $linear$, $PreLU$, and $softplus$.

Loss function and Optimization Function

Model training involves the use of loss and optimization functions. In order to optimize the search space of AutoCDRP to fit graphs with different distributions more effectively, we utilize various widely used loss and optimization functions. There are several options for loss functions, such as CrossEntropyLoss and NllLoss, as well as options for optimization functions, such as Adam and SGD.

Pooling Function.

The global pooling function is used to aggregate the node representations throughout the entire graph in order to obtain the graph-level representation from the node-level representations. As part of the proposed search space, graph pooling options include $GlobalAddPooling$ and $GlobalMaxPooling$.

Dropout and other hyper-parameters

With dropout operation, over-fitting can be prevented during the training process. In the proposed search space, dropout operation options include 0, 0.2, 0.4, and 0.6. Similarly, we provide options for learning rate and L2 regularization in the search space. The options for learning rate include 0.01, 0.001, 0.0001, and 0.0005, while options for L2 regularization include 0.001, 0.0001, and 0.0005.

5 Experiment Setup

Baseline Methods. In this study, we compare the GNN architectures output by AutoCDRP with tCNNS (Liu *et al.*, 2019) and GraphDRP (Nguyen *et al.*, 2021). tCNNS is a convolutional neural networks-based method where drugs are represented as SMILES string. In tCNNS a convolution layer is used to extract drug features from SMILES format and another convolution layer is used to extract cancer cell line features from genetic attribute vectors. Finally fully connected is added to the drug-cell response. GraphDRP is a graph neural network-based model where drugs are represented as graphs and cell-line as one-hot vectors. In GraphDRP, drugs and cell-line feature representations are learned by graph convolutional layers and 1D convolutional layers, respectively. Then, the concatenation of drug

and cell-line representation is used to predict the IC50 value. GraphDRP uses four variants of graph neural networks for learning features of drugs including GIN, GAT, GCN, and GAT with GCN. we denote the model obtained with each of them by GraphDRP_GIN, GraphDRP_GAT, GraphDRP_GCN, and GraphDRP_GAT-GCN, respectively.

Implementation Settings. To train the surrogate model, we sample 800 architectures and select k=100 best-predicted graph neural architectures for validation. Throughout all steps, the number of epochs was set to 20, except when evaluating the best architecture, where 300 is used instead. We predict the performance of 10^6 architectures.

A graph isomorphism network model with two layers is used as a surrogate model. A random 80%/20% split is chosen for the dataset split. Surrogate models take architectural graphs as inputs and predict the RMSE of architectures as scalar vectors. When the element-wise error is below $\beta = 1$, a squared term is used for loss calculation, and otherwise a mean absolute term. The Adam optimizer is used to update architecture weight. The learning rate and $L2$ regularization values are 0.00001 and 0.0005, respectively. In the experiments, the surrogate model is trained for 500 epochs. The experiments are implemented using Pytorch-Geometric library[1] (Fey and Lenssen, 2019)

Evaluation Metrics. In order to evaluate both the performance of architectural models and the surrogate model, Pearson correlation coefficients (PCCs) and root mean square errors (RMSEs) are used. As a bivariate statistical model, Pearson correlations measure the strength of linear relationships between two variables. An absolute negative linear correlation of -1 indicates a perfect negative correlation, a constant negative correlation of zero, and a perfect positive correlation of one indicates a perfect positive correlation. A Pearson correlation can be expressed as follows:

$$PCC = \frac{\sum_{i}^{n}(o_i - y_i)^2}{\sigma O \sigma Y}$$

where O and Y represent the set of true ln(IC50) and predicted ln(IC50), respectively. n is the number of data points, o_i and y_i are the true ln(IC50) and the predicted ln(IC50) of the ith data point, respectively.

The root mean square error (RMSE) is a standard method for estimating the error in predicting quantitative data given a model. Based on the number of data points, a true ln(IC50) o_i of the sample, and a predicted ln(IC50) y_i of the sample, the RMSE is defined as follows:

$$RMSE = \sqrt{\frac{1}{n}\Sigma_{i=1}^{n}(o_i - y_i)^2}$$

In addition, we use Kendall tau correlation to evaluate the degree of concordance between the predicted performance evaluation and the ground truth performance evaluation. Kendall tau correlation can range from +1 to -1. As a result, a perfect linear relationship will yield a correlation coefficient of 1 and no linear relationship will yield a correlation coefficient of 0. In addition, Kendall's tau correlation has a low overall error sensitivity and asymptotic variance. It can be defined as follows:

$$\tau = \frac{d}{n(n-1)/2}$$

where n is the number of data points and d is the difference between the number of concordant (ordered in the same way) and discordant (ordered differently) pairs.

6 Designed architecture by AutoCDRP

The best architectures found by AutoCDRP are reported in table 2.

7 Statistical test

We apply nonparametric tests that are known to be suitable for comparing predictive models based on multiple data samples (Demsar, 2006; García *et al.*, 2010). Compared to the parametric tests, the nonparametric tests require fewer assumptions (Demsar, 2006). In this analysis, we use both GDSC

[1] https://github.com/rusty1s/pytorch_geometric

Table 2: Designed architectures by AutoCDRP.

Component	Mixed test	Unseen drugs test	Unseen cells test
**Aggregation layer 1**	mean	max	max
**Convolution layer 1**	linear	GENConv	GENConv
**Activation layer 1**	PReLU	relu	PReLU
**Normalizer layer 1**	BatchNorm	BatchNorm	BatchNorm
**Multi-head layer 1**	1	1	1
**Aggregation layer 2**	add	add	add
**Convolution layer 2**	GENConv	GENConv	GENConv
**Activation layer 2**	relu	PReLU	PReLU
**Normalizer layer 2**	BatchNorm	None	BatchNorm
**Multi-head layer 2**	1	2	8
**Hidden dim**	64	64	256
**Dropout**	0.5	0.4	0.2
**Learning rate**	0.0001	0.005	0.0001
**Weight regulation**	0.0001	0.0001	0.0001
**Optimizer**	adam	adam	adam
**Loss function**	MSELoss	MSELoss	MSELoss
**Pooling function**	global_max_pool	global_max_pool	global_max_pool

and CCLE datasets and consider all experiments including mix test (GDSCMix and CCLEMix), blind test with unseen drugs (GDSCBD and CLLEBD), and blind test with unseen cell lines (GDSCBC and CCLEBC) experiments for each dataset. Thus, we make the analysis with height methods and six datasets presented in Table 3. We use the Friedman ranking test(García *et al.*, 2010) to access the statistical significance of differences in the model's performance over the datasets, based on the Pearson correlation coefficient (PCC). The overall performance ranking of compared methods to the PCC metric is presented in Figure 1. It can be seen that the proposed AutoCDRP ranks first.

Table 3: Dataset and methods used for the statistical test. Extracted from overall performance comparison table

	GDSCMix	CCLEMix	GDSCBD	CCLEBD	GDSCBC	CCLEBC
tCNNS	0.916	0.772	0.0617	0.0226	0.349	0.2027
GCN	0.9118	0.7858	0.4467	0.3769	0.823	0.7279
ARMA	0.9129	0.7826	0.3895	0.0327	0.8325	0.7287
ChebNet	0.9135	0.7822	0.31	0.3162	0.836	0.7285
GraphDRP	0.93	0.7834	0.4292	0.3687	0.8302	0.7292
GraOmicDRP	0.9284	0.7556	0.4126	0.0541	0.8376	0.7252
GraTransDRP	0.93	0.7736	0.5269	0.0616	0.8405	0.7257
AutoCDRP	0.9476	0.7886	0.5271	0.3862	0.8632	0.7332

Then, we obtain family-wise p-values with Bonferroni correction. We calculate the chi-square as shown in Eq. 1,

$$\mathcal{X}_F^2 = \frac{12N}{k(k+1)}[\sum_{j=1}^{k} R_j^2 - \frac{k(k+1)^2}{4}] \tag{1}$$

where N is the number of datasets, k is the number of methods and R_j the average ranking of the jth method. With N=6 and k=8, we can get $\mathcal{X}_F^2 = 23.7351$ Next, we calculate F_F as shown in Eq. 2.

$$F_F = \frac{(N-1)\mathcal{X}_F^2}{N(k-1) - \mathcal{X}_F^2} \tag{2}$$

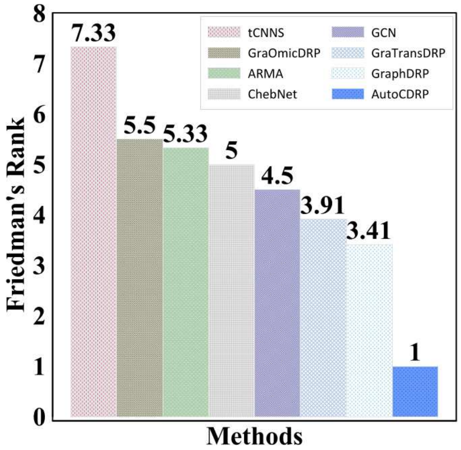

Figure 1: Average Friedman ranking values of AutoCDRP and baseline methods. AutoCDRP ranks first.

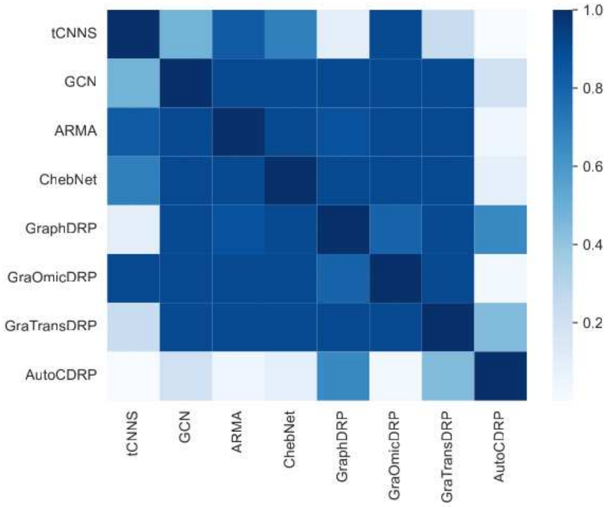

Figure 2: Nemenyi test scores. A low score means a significant difference between the performance of methods

In this experiment with height algorithms and six datasets, $F_F = 6.497$. F_F is distributed according to the F-distribution with $8 - 1 = 7$ and $(8 - 1) \times (6 - 1) = 35$ degrees of freedom. According to the F-distribution table, the critical value (CV) of F_F is 1.89 for $\alpha = 0.1$. As a result, the hypothesis of "equal" performance among compared methods is clearly rejected as $F_F > CV$.

Consequently, we use the Bonferroni correction (Holm, 1979) as a post-hoc test to compute family-wise p-values. In this computation, we set AutoCDRP as the control method and compute the family-wise p-values. The family-wise p-values between AutoCDRP and baseline methods are 7.5224e-06, 0.0014, 0.0021, 0.0046, 0.0133, 0.0391, 0.0874 for tCNNS, GraOmicDRP, ARMA, ChebNet, GCN, GraTransDRP, and GraphDRP, respectively. It is revealed from the family-wise p-values that PG-NAS shows significantly better performance over existing graph neural architecture search baseline frameworks with $\alpha = 0.1$.

Finally, we make the Nemenyi test (Demsar, 2006) to see how other baseline methods perform against each other. The Nemenyi test shows that every baseline method has significantly better performance over at most one other method. Figure 2 shows that every baseline method has significantly better performance over at most one other method, which confirms the superiority of PGNAS among compared methods.

References

Demsar,J. (2006) Statistical comparisons of classifiers over multiple data sets. *J. Mach. Learn. Res.,* **7**, 1–30.

Fey,M. and Lenssen,J.E. (2019) Fast graph representation learning with pytorch geometric. *CoRR,* **abs/1903.02428**.

García,S., Fernández,A., Luengo,J. and Herrera,F. (2010) Advanced nonparametric tests for multiple comparisons in the design of experiments in computational intelligence and data mining: experimental analysis of power. *Information sciences,* **180** (10), 2044–2064.

Holm,S. (1979) A simple sequentially rejective multiple test procedure. *Scandinavian journal of statistics,* **6**, 65–70.

Liu,P., Li,H., Li,S. and Leung,K.S. (2019) Improving prediction of phenotypic drug response on cancer cell lines using deep convolutional network. *BMC bioinformatics,* **20** (1), 1–14.

Nguyen,T., Nguyen,G.T., Nguyen,T. and Le,D.H. (2021) Graph convolutional networks for drug response prediction. *IEEE/ACM transactions on computational biology and bioinformatics,* **19** (1), 146–154.
